# Evaluation of a voluntary nutritional information program versus calorie labelling on menus in Canadian restaurants: a quasi-experimental study design

**DOI:** 10.1186/s12966-019-0854-x

**Published:** 2019-10-25

**Authors:** Lana Vanderlee, Christine M. White, David Hammond

**Affiliations:** School of Public Health and Health Systems, University of Waterloo, 200 University Ave W, Waterloo, ON N2L 3G1 Canada

**Keywords:** Menu labelling, Calorie labelling, Nutrition information, Restaurants

## Abstract

**Background:**

A significant proportion of the Canadian diet comes from foods purchased in restaurant settings. In an effort to promote healthy eating, the province of British Columbia (BC) implemented the *Informed Dining Program* (IDP), a voluntary, industry supported information program in 2012, while the province of Ontario implemented mandatory calorie labelling on menus in 2017. The study examined differences in awareness and the self-reported influence of nutrition information on food choices in restaurants with voluntary nutrition information, calorie labelling on menus, and no nutrition information program.

**Methods:**

Exit surveys were conducted outside of nine chain restaurants in Toronto, Ontario and Vancouver, British Columbia (Canada) in 2012, 2015, and 2017 with varying nutrition information programs implemented. Logistic regression analyses compared self-reported noticing and influence of nutrition information in restaurants with: 1) the IDP which provided nutrition information upon request, 2) calorie labelling on menus, and 3) control restaurants with no specific nutrition information program in place, adjusted for year, city and socio-demographic characteristics. Awareness and knowledge of the IDP were also examined.

**Results:**

There were no significant differences in noticing and self-reported influence of nutrition information on food choices between restaurants with the IDP and restaurants with no program. Participants were more likely to notice nutrition information in restaurants when calorie information was provided on menus (57%) compared to in restaurants with the IDP (22%, AOR = 6.20, 95%CI 3.51–10.94, *p* < 0.001) or restaurants with no nutrition information program (20%, AOR = 7.44, 95%CI 4.21–13.13, *p* < 0.001). Participants in restaurants with menu labelling were also more likely to report that nutrition information influenced their food purchase (38%) compared to restaurants with the IDP (12%, AOR = 4.43, 95%CI 2.36–8.30, *p* < 0.001) and restaurants with no nutrition information program (12%, AOR = 5.29, 95%CI 2.81–9.95, *p* < 0.001). Fewer than 1 in 5 participants who visited an IDP restaurant had heard of the IDP across all data collection years in both cities.

**Conclusions:**

There was no evidence that voluntary programs which provide nutrition information upon request were effective. Providing calorie information on menus increased the likelihood that consumers noticed and that their food choices were influenced by nutrition information in restaurant settings.

## Introduction

Canadians spend approximately 30% of their food budget on meals purchased in restaurant settings [1, 2]. Given the significant contribution of meals outside of the home to Canadian diets, helping consumers to make healthier food choices when dining out is paramount to improving overall diet quality.

**Fig. 1:**
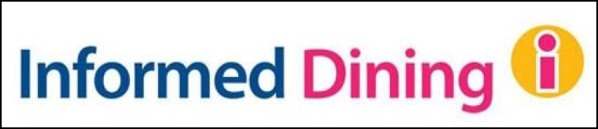
Informed Dining Program logo

Providing nutrition information in restaurants is one method to assist consumers in making informed food choices when they are eating away from home [3]. In 2017, the United States (US) implemented federal rule that requires major chains to post calorie information on menus or menu boards as part of the *Affordable Care Act* [4]. No federal policy has been implemented in Canada; however, the province of Ontario implemented the *Healthy Menu Choices Act* in 2017*,* which required chain restaurants with more than 20 outlets in the provinces to provide calorie information on menus [5]. In all other Canadian provinces and territories, nutrition information is provided by restaurants on a voluntary basis. In 2012, the Informed Dining Program (IDP) was developed by the Government of British Columbia, in collaboration with members of the food industry and non-governmental organizations, to standardize the information voluntarily provided in restaurant settings [6]. Food service establishments that opt into the program are required to display the program logo (see Fig. 1) and a statement on their menu or menu board indicating that nutritional information is available upon request. Information is provided for calories and the 13 core nutrients shown in the Nutrition Facts table in Canada. The IDP was first implemented in British Columbia in 2012, and was rolled out Canada-wide among select national chain restaurants in 2013 onwards. In 2016, 15% of all restaurants in British Columbia were participating in the IDP, including 45% of chain restaurants [7]. Comprehensive evaluations of the IDP to date have only been conducted in British Columbia, and have not been independently conducted [7]. Despite the intention of the IDP to standardize the provision of nutrition information in restaurants in Canada, a 2015 study on the availability nutrition information in the top 10 fast-food chain restaurants in Canada showed that, while 96% of restaurants had nutrition information available in some format, the information was available sporadically throughout restaurants in a variety of locations [8].

There is a need to examine the effectiveness of structured voluntary, industry-based programs compared to mandatory calorie labelling policies that require nutrition information to be posted on menus. To date, there has been little evaluation of how the availability and presentation of nutrition information has influenced consumer noticing and influence in restaurant settings. The implementation of the IDP and calorie labelling on menus in Canadian provinces provided an opportunity to evaluate a program that makes nutrition information available upon request (the IDP) compared to information immediately available on restaurant menus, using a quasi-experimental study design. The objective of the current study was to evaluate the impact of the IDP on consumers noticing and being influenced by nutrition information in restaurant settings compared to mandatory calorie labelling on restaurant menus and menu boards, and to examine changes in awareness of the IDP over time.

## Methods

**Fig. 2:**
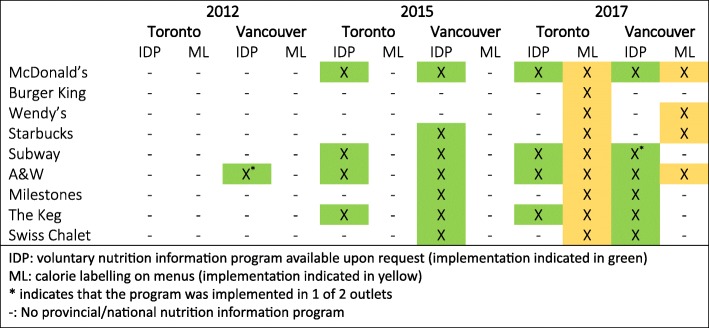
Implementation of the Informed Dining Program (IDP) and Menu Labelling (ML)

The study used a quasi-experimental design to examine changes in consumer noticing and influence of nutrition information in restaurants in Vancouver, British Columbia and Toronto, Ontario before and after changes to policies occurred as part of a larger, international study on menu labelling in Canada and the US. Exit surveys were conducted in September–November of 2012, 2015 and 2017 with restaurant patrons outside six quick-service restaurant chains (McDonalds, Burger King, Wendy’s, Starbucks, Subway and A&W) and three sit-down restaurants (Milestones, The Keg and Swiss Chalet) outside a total of 52 individual restaurants with varying nutrition information interventions in each jurisdiction over time. As shown in Fig. 2, in 2012, the voluntary IDP was implemented in one chain where participants were surveyed in Vancouver, and there was no nutrition information program in restaurants in Ontario. In 2015, the IDP was voluntarily implemented in some restaurant chains in Canada at a national level, such that 7 of 9 restaurant chains surveyed in Vancouver and 4 of 9 restaurants chains surveyed in Toronto had the program. In 2017, there were no required changes to policy in Vancouver; however 4 of 9 chains voluntarily added calorie labelling on menus (likely a result of the mandatory calorie labelling regulation in Ontario); while in Toronto, mandatory calorie labelling on menus of all chain restaurants came into force.

In each city, surveys were conducted at two or three outlets per restaurant chain, in different neighbourhoods to minimize potential bias due to neighbourhood socioeconomic status. Research assistants conducted environmental scans in each restaurant outlet to verify whether the IDP and calorie labelling on menus was present at restaurants where surveys were conducted. No personally identifying information was collected, and all data were anonymized.

Exit surveys were conducted daily during lunch and dinner hours in September, October and November. Restaurant patrons were approached upon exiting the restaurant using an intercept method and invited to participate in a 10-min survey on food choices in restaurants. Interviewer-assisted surveys were administered using iPads. Participants received $5 CAD as remuneration for their time. Individuals were eligible to participate if they were 18 years or older, had purchased food or drinks at the restaurant prior to completing the interview, and had not previously participated in the study. At sit-down restaurants, those who purchased take-out were not eligible. Verbal informed consent was obtained from all participants prior to completing the survey. Response rates for the 2012, 2015 and 2017 surveys were 22, 15, and 14%, respectively, based on AAPOR response rate #4 [9]. This study received ethics clearance through the Office of Research Ethics at the University of Waterloo (ORE # 18298).

Sample size was calculated for the larger international study, for which a sample size of 1000 survey participants in four “paired” cities with and without a menu labelling provide 80% power to detect a 6.5% difference between conditions for a 2-sided t-test, where α = .05.

### Survey measures

#### Nutrition information in restaurants

Participants were asked if they had noticed any nutrition information in the restaurant, and if so, where the information was located, what type of information they noticed, and when they noticed the information (before, during or after ordering). For each location where participants indicated they had noticed nutrition information, they were asked if the information in that location influenced what they ordered (‘food purchase’), and if so, how it had influenced their food purchase. These measures were adapted from previously published research [10].

In all 3 years of data collection in Vancouver, and in 2015 and 2017 in Toronto, participants were asked if they had ever heard of the IDP. If they had, participants were asked to describe the program; responses were coded by the interviewer as correct if they mentioned something related to nutrition information for restaurant food (either on the internet or in the restaurant). In 2015 and 2017, if participants had correctly described the program, they were also asked if they had ever used the nutrition information provided by the IDP, and if the restaurant they had visited was part of the IDP. Lastly, participants were asked where they had seen or heard about the IDP.

#### Perceptions of nutrition information availability

Participants were asked to rate on a scale from 1 to 10 how easily available nutrition information is in restaurants in general, as well as how easily available nutrition information was at the restaurant that they had visited.

#### Socio-demographics measures

Socio-demographic questions included sex, age, education, household income, and race (White or other ethnicity). Self-reported height and weight were used to calculate body mass index (BMI), categorized according to WHO categories [11]. A programming error resulted in the loss of unsaved open-ended information for some participants in 2015 for race, age, height and weight data (*n* = 788). These participants were maintained in the sample, and a categorical variable for ‘Don’t know / Refused / Missing’ was included accordingly, where possible.

### Analysis

Descriptive analyses were used to describe the frequency of noticing information overall and at specific locations within the restaurant (menu/menu board, wall/window/door, on a poster, pamphlet, on the item, tray liner, next to item, computer/kiosk, other). Descriptive analyses also examined when information was noticed (before/during ordering, or after ordering), and what type of information was noticed (calories, fat, sugar/carbohydrates, sodium/salt, health logo/symbol, allergen, vegetarian, organic, other). Logistic regression models were fitted to examine the likelihood of noticing any nutrition information overall, noticing information at each location within the restaurant, noticing each type of nutrition information, noticing before or during ordering, and the influence of nutrition information on food purchase (0 = no, 1 = yes), including variables for labelling intervention (none, IDP, or calorie labelling), city (Toronto, Vancouver), year (2012, 2015, 2017), restaurant chain, and socio-demographics (gender (male/female), education (high school or less, some additional training, higher education), income quartile (low, low to moderate, moderate to high, high, not stated), race (White, Other, Refused), and BMI (< 18.5, 18.5–24.9, 25–29.9, 30+, Not stated)). Sensitivity analysis determined that age was not associated with noticing or influence of nutrition information in 2012 and 2017, and thus, age was not included in the models.

Separate logistic regression models were used to examine awareness and correctly describing the IDP (0 = no, 1 = yes) each stratified by city given the varying timeline for implementation between the cities. The models used data from all years in Vancouver, and 2015 and 2017 in Toronto, adjusting for year, restaurant chain, as well as socio-demographic covariates (sex, education, income quartile, race and BMI).

Lastly, linear regression models were constructed to examine differences in the perception of the availability of nutrition information in general and at the restaurant where the participant was surveyed, adjusting for city, year, and socio-demographics.

## Results

The overall sample size was 5197; however, a small proportion (< 2%) of participants did not report data for key demographic factors (i.e., education and gender) and were excluded from the sample. A total of 1413 participants were recruited in 2012, 2217 in 2015 and 1423 in 2017 for an overall analytical sample of 5053, 53.4% of which were from Toronto (*n* = 2698) and 46.6% were from Vancouver (*n* = 2355). Sample characteristics of the final analytical sample are shown in Table 1.

**Table 1:** Sample characteristics (*N* = 5053)

	Overall (*N* = 5053)	Calorie labelling on menus (*n* = 1244)	Informed Dining program (*n* = 1533)	No labelling (*n* = 2276)	X^2^ (*p*-value)
	% (n)	% (n)	% (n)	% (n)	
Gender					12.1 (*p* = 0.002)
Male	61.8% (3125)	63.1% (785)	58.3% (893)	63.6% (1447)	
Female	38.2% (1928)	36.9% (459)	41.7% (640)	36.4% (829)	
Age group					321.22, (*p* < 0.001)
18–29 years	35.3% (1784)	40.1% (499)	28.0% (429)	37.6% (856)	
30–49 years	32.5% (1630)	32.6% (405)	28.6% (438)	34.6% (787)	
50 + years	23.6% (1192)	35.7% (320)	34.5% (376)	21.8% (496)	
Not stated / missing	8.8(447)	1.6% (20)	18.9% (290)	6.0% (137)	
Education level					23.73 (*p* < 0.001)
High school or less	27.4% (1383)	26.8% (334)	23.8% (365)	30.1% (684)	
Some additional training	27.5% (1390)	27.1% (337)	27.2% (417)	27.9% (636)	
Higher education	45.1% (2280)	46.1% (573)	27.9% (636)	42.0% (956)	
Income quartile					37.35 (*p* < 0.001)
Low	21.9% (1106)	22.5% (279)	19.0% (292)	23.5% (535)	
Low to moderate	19.8% (998)	18.8% (234)	19.5% (299)	20.4% (465)	
Moderate to high	21.3% (1074)	22.6% (281)	22.1% (339)	19.9% (454)	
High	22.2% (1120)	22.1% (275)	26.0% (399)	19.6% (446)	
Not stated	14.9% (755)	14.1% (175)	13.3% (204)	16.5% (376)	
Race					20.59 (*p* < 0.001)
White	58.7% (2966)	54.9% (683)	60.7% (931)	59.4% (1352)	
Other	39.0% (1970)	43.5% (541)	36.2% (555)	38.4% (874)	
Not stated / Missing	2.3% (117)	1.6% (20)	3.1% (47)	2.2% (50)	
BMI					231.87 (*p* < 0.001)
Underweight	2.9% (147)	3.1% (39)	2.5% (38)	3.1% (70)	
Normal weight	41.5% (2097)	46.3% (576)	35.7% (547)	42.8% (974)	
Overweight	27.0% (1365)	30.5% (380)	23.8% (365)	27.2% (620)	
Obese	12.9% (654)	14.5% (180)	11.8% (181)	12.9% (293)	
Not stated	15.6% (790)	5.5% (69)	26.2% (402)	14.0% (319)	

### Noticing nutrition information

**Fig. 3:**
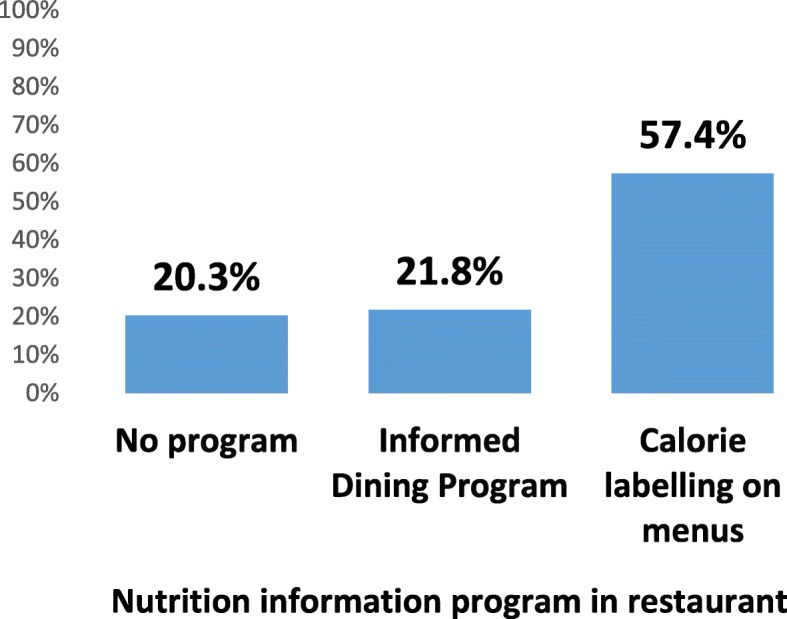
Percentage of restaurant patrons who noticed any nutrition information during their visit, by nutrition information program (*n* = 5053)

Figure 3 shows the percentage of participants who reported noticing nutrition information, according to the type of nutrition information program present in the restaurant (no program, IDP only, or calorie labelling on menus whether or not the restaurant was part of the IDP).

In regression models, participants at restaurants with calorie labelling on menus were significantly more likely to notice nutrition information than those at an IDP restaurant (AOR = 6.20, 95%CI 3.51–10.94, *p* < 0.001) or at a restaurant with no nutrition information program (AOR = 7.44, 95%CI 4.21–13.13, *p* < 0.001). There was no significant difference in noticing nutrition information between patrons at IDP restaurants and patrons at restaurants with no program (AOR = 0.83, 95%CI 0.65–1.07, *p* = 0.14). There was a significant effect of year, whereby after adjusting for the nutrition information program in restaurants, participants were significantly more likely to notice information in 2012 than in 2015 (AOR = 1.31, 95%CI 1.05–1.63, *p* = 0.02). There was also a significant effect of city, such that compared to Toronto, those in Vancouver were less likely to notice nutrition information overall (AOR = 0.78, 95%CI 0.68–0.90, *p* = 0.001).

**Fig. 4:**
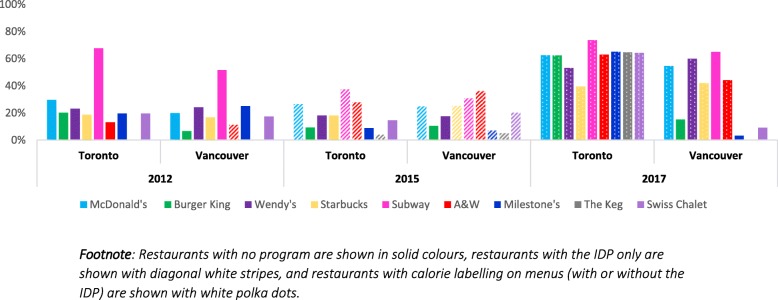
Percentage who reported noticing nutrition information during their visit, by restaurant and city, in each year

Noticing was significantly different between restaurants chains, after adjusting for the nutrition information program in restaurants (see Fig. 4). Although several significant contrasts between restaurants were observed, participants were consistently more likely to notice nutrition information at Subway restaurant compared to all other restaurants (*p* < 0.001 for all contrasts). No differences in noticing nutrition information were observed for sex, education, income quartile, race or BMI.

Table 2 shows the locations within the restaurant where participants reported noticing nutrition information. After adjusting for city, year, restaurant, and sociodemographic differences, participants were more likely to see nutrition information on menus when there was calorie labelling on menus compared to restaurants with the IDP (AOR = 0.071, 95%CI 0.033–0.153, *p* < 0.001) or no nutrition information program (AOR = 0.060, 95%CI 0.027–0.133, *p* < 0.001), with no differences between the IDP or no nutrition information program. There were no other significant differences between nutrition information programs for other locations inside the restaurant.

**Table 2:** Locations of information noticed, according to the type of nutrition information program present in the restaurant (*n* = 5053)^a^

	No program (*n* = 2276)	Informed Dining Program (*n* = 1533)	Calorie labelling on menus (*n* = 1244)	X^2^ statistic^b^, *p*-value
Menu/menu board	7.6%	7.1%	46.6%	49.9, *p* < 0.001
Wall/window/door	4.0%	3.5%	4.0%	0.2, *p* = 0.91
On a poster	3.3%	8.9%	4.2%	2.7, *p* = 0.26
Pamphlet	1.7%	2.5%	1.3%	1.1, *p* = 0.57
On the item	1.6%	2.3%	1.0%	1.2, *p* = 0.54
Tray liner	1.9%	2.5%	1.0%	0.9, *p* = 0.63
Next to item	2.0%	1.4%	3.3%	2.0, *p* = 0.36
Other	1.1%	1.2%	0.6%	0.5, *p* = 0.77
Computer/kiosk	0.7%	0.7%	1.4%	0.03, *p* = 0.99

^a^Note: Participants could report more than one location. Reported among all participants

^b^Chi-square statistic for the nutrition information program variable in logistic regression models for each location, adjusted for city, year and socio-demographic variables

As shown in Table 3, calories were the most commonly reported type of nutrition information noticed, regardless of the type of nutrition information program in the restaurant. Participants were significantly more likely to notice calorie information at restaurants where calorie labelling was present on menus compared to at restaurants with the IDP (AOR = 6.70, 95%CI 3.41–13.15, *p* < 0.001) or no nutrition information program (AOR = 9.06, 95%CI 4.63–17.75, *p* < 0.001), with no differences between the IDP and no nutrition information program. There were no other differences in the types of information noticed between the nutrition information programs.

**Table 3:** Types of nutrition information noticed, according to the type of nutrition information program present in the restaurant^a^

	No program (*n* = 2276)	Informed Dining Program (*n* = 1533)	Calorie labelling on menus (*n* = 1244)	X^2^ statistic^b^, *p*-value
Calories	11.0%	11.7%	53.1%	41.6, *p* < 0.001
Fat	6.2%	5.6%	3.0%	1.24, *p* = 0.54
Sugar / Carb	1.9%	2.9%	1.8%	0.58, *p* = 0.75
Sodium / Salt	2.1%	2.0%	1.2%	1.67, *p* = 0.43
Health logo / Symbol	1.2%	1.0%	1.5%	0.02, *p* = 0.99
Allergen	1.1%	1.5%	1.1%	0.21, *p* = 0.90
Vegetarian	1.4%	1.3%	0.6%	0.96, *p* = 0.62
Organic	0.2%	0.6%	0.6%	0.38, *p* = 0.87
Other	4.3%	5.3%	3.1%	2.86, *p* = 0.24

^a^Note: Participants could report more than one type of information noticed. Reported among all participants

^b^Chi-square statistic for the nutrition information program variable in logistic regression models for each location, adjusted for city, year and socio-demographic variables

In restaurants with calorie labelling, significantly more participants (50.6%) noticed nutrition information before or during their order compared to in IDP restaurants (16.2%) (AOR = 6.64, 95%CI 3.57–12.33, *p* < 0.001), or in restaurants with no program (15.2%) (AOR = 8.41, 95%CI 4.50–15.69, *p* < 0.001). There were no significant differences in when nutrition information was noticed between the IDP and restaurants with no nutrition information program.

### Influence of nutrition information on food purchase

**Fig. 5:**
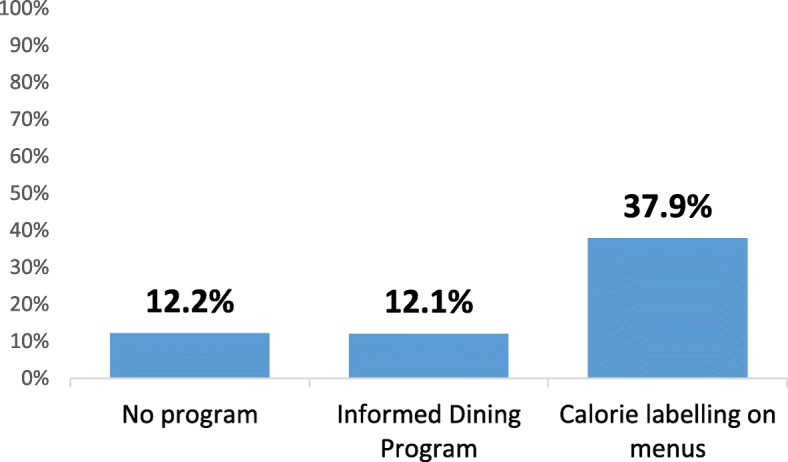
Percentage of the sample who reported nutrition information influenced their food purchase, according to the type of nutrition information program present in the restaurant, across all years (*n* = 5053)

Figure 5 shows the proportion of participants who reported that nutrition information influenced their food purchase, by type of nutrition information program, across all years.

In adjusted regression models, participants at restaurants with calorie labelling were significantly more likely to report that nutrition information influenced their food purchase than participants at restaurants with the IDP (AOR = 4.43, 95%CI 2.36–8.30, *p* < 0.001) and no nutrition information program (AOR = 5.29, 95%CI 2.81–9.95, *p* < 0.001). There was no significant difference in reported influence between participants at restaurants with the IDP and at restaurants with no program (AOR = 1.20, 95%CI 0.89–1.61, *p* = 0.24)). After adjusting for type of nutrition information program, city was significant in the model, whereby those in Vancouver were significantly less likely to report that the nutrition information influenced their food purchase, compared to those in Toronto (AOR = 0.75, 95%CI 0.64–0.88, *p* < 0.001). Restaurant type was significant in the model, with notable differences in reported influence at Subway compared to other restaurants (*p* ≤ 0.001 for differences between Subway and all other restaurants). There were no significant differences in reported influence by year or socio-demographic covariates.

### Awareness of the informed dining program

Awareness of the IDP program was stratified by city given the different timelines for implementation between the cities and measures used across years. In Vancouver, there were significant differences in the percentage of participants who had heard of the IDP between years: participants were more likely to have heard of the IDP in 2015 (18.4%) (AOR = 1.35, 95%CI 1.02–1.80, *p* = 0.04) and 2017 (19.7%) (AOR = 1.46, 95%CI 1.09–1.95, *p* = 0.01) compared to 2012 (14.4%). There was no difference in having heard of the IDP between 2015 and 2017. Respondents in Toronto were not asked about the IDP in 2012, and there was no significant difference in having heard of the IDP between 2015 (13.8%) and 2017 (16.2%) in Toronto.

In Vancouver, there were no changes in the percentage of participants who correctly described the program between years (10.8% in 2012; 12.4% in 2015; 12.7% in 2017). In Toronto, significantly more participants correctly described the program in 2017 (11.9%) compared to 2015 (9.5%) (AOR = 1.40, 95%CI 1.03–1.91, *p* = 0.03).

In 2015 and 2017, participants in both cities were asked if they had heard of the IDP, could describe the program, and then were asked if the restaurant that they had visited was part of the program. A total of 3.8% of participants (*n* = 136) had heard of the program, correctly described it, and knew whether or not it was in the restaurant they had visited.

The most commonly reported medium through which participants had heard of the IDP was in a restaurant (3.9%), on TV (2.1%), on the internet (2.1%) or from other people (1.2%). When asked specifically about the IDP program, 3.5% of the 2015 and 2017 samples (or 43.7% of those who were aware of the IDP program) reported that they had used the IDP nutrition information. (Note, this question was not asked in 2012).

### Perceived availability of nutrition information

Participants reported their perceived availability of nutrition information in restaurants in general (in all years) and for the specific restaurant where the participant was surveyed (in 2015 and 2017), on a scale from 1 (not at all easy to access) to 10 (extremely easy to access). When asked about restaurants in general, there was a significant effect of city and year, whereby participants in Vancouver perceived nutrition information as less available than those in Toronto (B = -0.48, 95%CI -0.62- -0.352), and participants perceived nutrition information to be less available in 2012 (B = -1.05, 95%CI -1.23 - -0.87) and 2015 (B = -1.12, 95%CI -1.29- -0.95) than in 2017, with no difference between 2015 and 2017.

When asked about the specific restaurant where the participant was surveyed in 2015 and 2017, perceived availability of nutrition information was greater in Toronto than Vancouver (B = 0.32, 95%CI 0.13–0.52, *p* = 0.001) and greater in 2017 than in 2015 (B = 1.92, 95%CI 1.72–2.12, *p* < 0.001).

## Discussion

Findings from the current study suggest there was little or no association between the Informed Dining Program, a voluntary program where nutrition information is provided upon request, and noticing and use of nutrition information in restaurant settings. Overall, awareness, knowledge and self-reported influence of the IDP was low, even after the program was rolled out Canada-wide, and the number of participating chains increased in both cities. Awareness of the program in this study in all years was slightly higher than a 2016 evaluation conducted by the Government of BC, which found that about 1 in 10 residents of BC were aware of the program, similar to rates in a 2013 evaluation following the May 2012 promotional campaign [7]. One would expect that awareness of the program would continue to increase over time as a result of increased exposure to the program in an increasing number of establishments, especially given that 45% of chain restaurants in BC participated in the program by 2016 [7]. While there was a small but significant increase from 2012 to 2015 in Vancouver, this increase diminished over time and overall rates of awareness remained low, with less than 1 in 5 participants in Vancouver having heard of the program. A very small proportion of the sample was aware of the program and could also correctly describe its content and whether or not it was in the restaurant they had just visited; again reinforcing that the reach of the program amongst those making purchases in restaurants is low.

There was a significant association between providing calorie labelling information directly on restaurant menus and menu boards and awareness and use of nutrition information in the restaurant setting. This is consistent with the majority of the menu labelling literature that has demonstrated greater rates of noticing and influence of nutrition information when it is provided on menus in a standardized format and visible immediately at the point-of-purchase compared to traditional restaurant practices [12–16]. Increased awareness of nutrition information in restaurants in Toronto may have been in part due to the recent implementation of the *Healthy Menu Choices Act* in 2017, which received a considerable amount of media attention; however, data collection occurred 10 months after implementation which would likely have dampened this effect. Overall, rates of noticing and influence on nutrition information in restaurants with calorie labelling on menus in this study were substantial.

The prevalence of noticing nutrition information in restaurants in this study was similar to previous studies. Self-reported data from a 2013/14 population-level telephone survey in the US suggests that 43.4% of Americans saw nutrition information in a fast-food restaurant in the past 6 months, and 32.8% saw it in a sit-down restaurant, and approximately half used that information to inform their food choice [17]. These estimates for noticing nutrition information are similar to the present study, considering a national US survey at that time would have captured jurisdictions with and without calorie labeling on menus.

Unsurprisingly, calorie information was noticed more frequently when it was posted directly on menu boards, with no differences in noticing other types of nutrition information that are provided by the IDP, such as sodium or sugars. Proponents of the IDP commend the program for providing more comprehensive nutritional information than calories alone, which is sometimes described as a reductionist approach to identifying healthy foods. However, the current results suggest that the IDP is no more effective at increasing attention to other nutrients.

The proportion of consumers who were influenced by the nutrition information to inform their food purchase was also similar to, though somewhat higher than, other studies. Previous research indicates that approximately one-third of restaurants patrons who notice nutrition information report using it. In the current study, the proportion of participants in the current study who reported that their food purchase was influenced by nutrition information was somewhat higher than use reported in other Canadian [10, 18–20] and international studies [12, 14, 21–26].

This study did not identify consistent socio-demographic differences in noticing and use of nutrition information, thus suggesting that a mandatory policy for calorie labelling on menus has an equitable effect and is unlikely to exacerbate current diet-related inequities. The overall literature has been inconsistent with regards to the differential impact that menu labelling may have on population sub-groups; the current study did not find any significant differences to report [13, 20, 26]. It should be noted that sociodemographic disparities may be lower when nutrition information is more readily accessible (i.e., on menus) compared to when information is available upon request, which may be selectively accessed.

This study also highlights the roles that individual restaurants can play in displaying nutrition information, irrespective of any voluntary or mandatory nutrition information programs or policies in place. For example, Subway had considerably higher levels of noticing and use of nutrition information than any other restaurants, and had noticing and use rates in 2012 similar to other restaurants in 2017 where mandatory nutrition information was posted on menus. In this instance, requiring mandatory labels reduced differences in noticing and use of nutrition information between Subway and other restaurants. This may relate to the type of food served in Subway restaurants (e.g., ‘healthier’ sandwich options) and the consumers who are seeking out potentially healthier fast-food alternatives; it also may relate to the marketing and branding that Subway commits to in order to address the desires of their customer base. Previous research has also indicated that there are differences in noticing, use and impact on food purchase between types of restaurants, even within quick-service restaurants, with ‘food chains’ being associated with a greater impact of menu labelling than ‘coffee chains’ [21, 26].

Interestingly, four national chain restaurants provided calorie labelling on menus in 2017 in both Toronto and Vancouver, which was not required according to any provincial regulations in Vancouver. This is similar to the period prior to national legislation in the US, in which several chains adopted mandated calorie labels at a national level after they were required in several states and cities prior to the implementation of the federal requirement for calorie labels under the Food and Drug Administration (FDA). The diffusion of calorie labelling practices in chain restaurants to provinces where no policy is in place is a potential unintended benefit of mandatory provincial-level menu labelling policy, and may suggest support from the food industry for national implementation of a menu labelling strategy.

### Limitations and strengths

The study may be susceptible to self-report or social desirability bias, as well as selection bias such that those who are more interested in nutrition may be more likely to participate in the study. However, any bias would be consistent between sites and over time and should not affect the direction of the results. The current study did not use probability-based sampling methods, and the sample had more males than females, was slightly younger, had higher education and lower BMI than the general population, and the proportion of participants who were not White was higher than national estimates in Canada. Lastly, while models were adjusted for time, there may be other secular effects at play that influence the likelihood of noticing and using nutrition information over time that cannot be accounted for in the modelling. The range of restaurant types and inclusion of restaurants from different neighbourhoods with varying demographic profiles in the sampling strategy increases the generalizability of the results. A large sample size and an innovative quasi-experimental design provides a rigorous comparison with comparable control groups over time.

## Conclusions

Providing nutrition information upon request in a structured, voluntary, industry-led program was not effective in increasing noticing and use of nutrition information in restaurant settings. Despite national voluntary implementation in major chains over a span of 5 years, awareness and use of the Informed Dining Program was low. Providing calorie information directly on menus resulted in substantial increases in the proportion of restaurant patrons that noticed and used nutrition information to guide their food choices. The substantial increases in noticing and use of nutrition information in restaurants with calorie information on menus suggests that consumers are more likely to use nutrition information when it is more easily accessible and salient. The additional effort required to seek out nutrition information in a scenario where customers are making decisions in a short timespan may pose too great a barrier. These results have important implications for policy-makers considering implementing a mandatory menu labelling policy.

## Data Availability

The datasets generated and/or analysed during the current study are available from the corresponding author on reasonable request.
